# Monitoring Effect of Human Papillomavirus Vaccines in US Population, Emerging Infections Program, 2008–2012

**DOI:** 10.3201/eid2109.141841

**Published:** 2015-09

**Authors:** Susan Hariri, Lauri E. Markowitz, Nancy M. Bennett, Linda M. Niccolai, Sean Schafer, Karen Bloch, Ina U. Park, Mary W. Scahill, Pamela Julian, Nasreen Abdullah, Diane Levine, Erin Whitney, Elizabeth R. Unger, Martin Steinau, Heidi M. Bauer, James Meek, James Hadler, Lynn Sosa, Suzanne E. Powell, Michelle L. Johnson, HPV-IMPACT Working Group

**Affiliations:** Centers for Disease Control and Prevention, Atlanta, Georgia, USA (S. Hariri, L.E. Markowitz, E.R. Unger, M. Steinau, S.E. Powell, M.L. Johnson);; University of Rochester School of Medicine and Dentistry, Rochester, New York, USA (N.M. Bennett, M.W. Scahill);; Yale School of Public Health, New Haven, Connecticut, USA (L.M. Niccolai, P. Julian, J. Meek, J. Hadler);; Oregon Health Authority, Portland, Oregon, USA (S. Schafer, N. Abdullah);; Vanderbilt University School of Medicine, Nashville, Tennessee, USA (K. Bloch, D. Levine);; California Department of Public Health, Richmond, California, USA (I.U. Park, E. Whitney, H.M. Bauer);; Connecticut Department of Health, Hartford, Connecticut, USA (L. Sosa)

**Keywords:** human papillomavirus, HPV, HPV vaccine, cervical precancer, cancer, cervical intraepithelial neoplasia, CIN, CIN2, CIN3, adenocarcinoma in situ, vaccine effect, vaccination, viruses, United States, Emerging Infections Program

## Abstract

In 2007, five Emerging Infections Program (EIP) sites were funded to determine the feasibility of establishing a population-based surveillance system for monitoring the effect of human papillomavirus (HPV) vaccine on pre-invasive cervical lesions. The project involved active population-based surveillance of cervical intraepithelial neoplasia grades 2 and 3 and adenocarcinoma in situ as well as associated HPV types in women >18 years of age residing in defined catchment areas; collecting relevant clinical information and detailed HPV vaccination histories for women 18–39 years of age; and estimating the annual rate of cervical cancer screening among the catchment area population. The first few years of the project provided key information, including data on HPV type distribution, before expected effect of vaccine introduction. The project’s success exemplifies the flexibility of EIP’s network to expand core activities to include emerging surveillance needs beyond acute infectious diseases. Project results contribute key information regarding the impact of HPV vaccination in the United States.

## Abstract 2

Methods for surveillance of cervical precancers and associated types were developed to monitor effect of HPV vaccination.

Human papillomavirus (HPV) vaccines are primarily designed to prevent HPV-associated cancers that typically occur years to decades after exposure to HPV-16 and -18. Three prophylactic HPV vaccines are available in the United States: bivalent, quadrivalent, and 9-valent vaccines. The bivalent vaccine protects against HPV-16 and -18, the most common oncogenic HPV types, which are responsible for ≈70% of HPV-associated cervical cancers and a large proportion of other HPV-related cancers (*1*). The quadrivalent vaccine also protects against HPV-16 and -18 and against HPV-6 and -11, two nononcogenic HPV types that cause genital warts and respiratory papillomatosis (*2*). The 9-valent vaccine also protects against HPV-6, -11, -16, and -18 and against 5 other oncogenic types: HPV-31, -33, -45, -52, and -58 (*3*).

Since June 2006, the Advisory Committee on Immunization Practices (ACIP) has recommended routine HPV vaccination of girls 11–26 or 12–26 years of age who have not previously been administered the quadrivalent vaccine (*4*). After licensure of the bivalent vaccine against HPV-16 and -18 in 2009, the ACIP guidelines for vaccination of women and girls were expanded to recommend quadrivalent or bivalent vaccine for protection against HPV types that can cause cancer. The 9-valent vaccine was licensed in 2014, and in February 2015, ACIP included it as one of 3 recommended HPV vaccines (*5*). To date, the quadrivalent vaccine accounts for almost all HPV vaccines distributed (*6*).

Postlicensure surveillance activities include a range of early, mid, and late biological outcomes for the timely monitoring of the effects of the vaccines in the population (*7*). In the United States, type-specific HPV infection and genital warts are being monitored in a variety of settings to evaluate the earliest evidence of vaccine effect, and HPV-associated cancers are monitored through the National Cancer Institute’s Surveillance, Epidemiology, and End Results program and the Centers for Disease Control and Prevention (CDC)–administered National Program of Cancer Registries, which cover the entire US population (*8*,*9*). 

Because of the slow natural history of HPV oncogenesis, the effect of vaccination on invasive cancers will not be evident for decades. Preinvasive cervical intraepithelial neoplasia 2 and 3 and adenocarcinoma in situ (together referred to as CIN2+), which are detected through routine screening, take less time to develop and were used as a surrogate for cervical cancer in vaccine trials. Real-world reductions in CIN2+ have been shown in countries with high vaccination coverage and catch-up programs for older persons and where it is possible to link data across population-based disease, screening, and vaccination registries (*10*–*13*). 

In the United States, population-level CIN2+ declines that are attributable to vaccination are more challenging to measure because of a lack of national screening registries and because CIN2+ diagnosis is affected by changes in screening recommendations that have been implemented since vaccine introduction. Historically, cervical cancer screening guidelines in the United States differed across organizations in regard to the age for initial screening and the frequency of screening; many organizations (e.g., the American Cancer Society) recommended screening begin at age 18 or the age of onset of sexual intercourse, whichever was first (*14*). However, over the past decade, screening guidelines have evolved to recommend cervical cancer screening begin at older ages. Currently, all guidelines recommend beginning screening at 21 years of age and that the intervals between screenings be longer than previously recommended, particularly if HPV-based co-testing is used (*15*). Furthermore, CIN2+ lesions are not reportable to public health authorities, except in New Mexico, nor monitored through most existing cancer registries. Therefore, precise determination of the number of women screened is difficult, which, together with changes in and gradual implementation of cervical cancer screening recommendations, makes it difficult to determine whether declines in CIN2+ diagnoses are attributable to vaccination or changes in screening utilization. Given these limitations, additional measures, such as characterizing HPV types associated with CIN2+ lesions and obtaining vaccination histories, are needed to evaluate vaccine-attributable reductions in CIN2+ incidence among the US population.

In 2007, five sites within the Emerging Infections Program (EIP) received funding to determine the feasibility of establishing a population-based surveillance system that could, in addition to monitoring overall CIN2+ trends, enable monitoring of trends in HPV type distribution in CIN2+ lesions among vaccinated and unvaccinated women with a diagnosis of CIN2+. Although the EIP network has traditionally focused on acute infectious diseases with typically short incubation periods, their extensive expertise and proficiency for enhanced surveillance and demonstrated ability to develop local infrastructure to support complex surveillance activities made the EIP network an ideal candidate for collaboration on developing this new system.

The 5 EIP US sites selected to participate in the pilot HPV-IMPACT project were in California, Connecticut, New York, Oregon, and Tennessee. Catchment areas comprised 8 contiguous cities in northwest Alameda County, California; New Haven County, Connecticut; Monroe County, New York; a contiguous region including Portland and most of Washington and Multnomah Counties, Oregon; and Davidson County, Tennessee. On the basis of the 2010 US census, the population of women (>18 years of age) ranged from ≈260,000 to 350,000 for the 5 catchment areas. The size of each catchment area was selected to maximize successful implementation of all elements of the system while allowing adequate precision for measuring trends over time. The objectives of the HPV-IMPACT project were to 1) conduct active population- and laboratory-based surveillance of CIN2+ diagnoses in women >18 years of age residing in defined catchment areas, 2) determine HPV types in CIN2+ lesions among women 18–39 years of age, 3) collect relevant clinical information and detailed HPV vaccination history for women 18–39 years of age, and 4) estimate annual rates of cervical cancer screening among the catchment area population.

Initial activities included establishment of advisory committees comprising key community members, such as health practitioners, anatomic pathologists, and public health authorities, to assist with the development and implementation of the new surveillance system. A variety of methods were used to systematically identify all local and remote histopathology laboratories that serve residents of the catchment areas: conducting surveys of family practitioners, gynecologists, and laboratories; contacting local cancer registries; and using telephone and other directories. The number of laboratories identified in each catchment area varied widely, from 4 local laboratories in New York to 29 laboratories within and outside the catchment area in Connecticut. Although some of the same large national reference laboratories served multiple catchment areas, EIP sites had to work with different regional offices and staff to establish reporting. 

This project was reviewed by the following agencies and determined to be exempt from institutional review board approval because the activity constitutes routine disease surveillance activity for disease control program and policy purposes: CDC; Public Health Division, Oregon Health Authority; Tennessee Department of Health; and the institutional review boards of Yale University; University of California, Berkeley; University of California, San Francisco; Vanderbilt University; Alameda County Medical Center; Kaiser Permanente; Unity Health System; University of Rochester Research Subjects Review Board; and Health Clinical Investigation Committee. The project was approved by the State of California Committee for Protection of Human Subjects. Informed consent was not required by any reviewing or approving institution.

Because CIN2+ reporting was not legally required or routinely performed in any of the participating sites and the legal authority to mandate disease reporting rests with the state, each EIP site investigated the possibility of mandated reporting in their jurisdiction. Connecticut was the first to make the necessary changes to enable statewide CIN2+ reporting through the EIP in 2008 (*16*), Tennessee followed with mandated reporting through the state cancer registry in 2009, Oregon made CIN2+ reportable statewide in 2013 (with retroactive reporting starting in 2008), and California worked with the Alameda County health officer to mandate prospective reporting as of 2013. New York did not pursue reporting mandates because of legislative restrictions and because the established strong support and good working relationships with each laboratory ensured completeness of voluntary reporting.

A protocol and case report forms were developed to standardize methodology for case ascertainment, specimen and data collection, and DNA typing methods. A centralized electronic case management system was created for data collection and maintenance. Each EIP site’s unique characteristics required use of different strategies to achieve project objectives, so the system was designed to maintain standards while accommodating site-specific requirements. Because classification systems and nomenclature for preinvasive cervical neoplasia are not standardized in the United States, a master list of possible diagnostic codes, terminology, and synonymic search terms was developed and provided to all reporting laboratories to standardize case finding. Reporting laboratories were asked to provide demographic information for patients (age, race/ethnicity, and health insurance status), along with histopathologic findings. Reports were deduplicated, anonymized, and entered into the project database.

Additional information was obtained for women 18–39 years of age who received a diagnosis of CIN2+. Reporting laboratories were requested to provide samples of archived specimens. At most sites, 1 laboratory agreed to process all specimens according to standard procedures, and prepared specimens were sent to CDC (Atlanta, GA, USA) for HPV DNA typing. Laboratory methods have been previously described (*17*). In brief, a representative block of the diagnostic tissue was provided by the laboratory and presence of a lesion was histologically verified at CDC. DNA was extracted and tested by using the Linear Array HPV Genotyping Assay (Roche Diagnostics, Indianapolis, IN, USA), which detects 37 HPV types (6, 11, 16, 18, 26, 31, 33, 35, 39, 40, 42, 45, 51, 52, 53, 54, 55, 56, 58, 59, 61, 62, 64, 66, 67, 68, 69, 70, 71, 7273, 81, 82, 83,84, 89, IS39). Samples with inadequate or HPV-negative assay results were retested by using the INNO-LiPA HPV Genotying Extra Assay (Innogenetics, Gent, Belgium). Samples negative for the genomic control probe and HPV by both assays were considered inadequate and omitted. HPV vaccination history was investigated by using a variety of sources and methods, as appropriate for each site. Information was collected regarding the number, date, and type of each vaccine dose received. Identified sources for vaccination history comprised state immunization registries, outpatient provider medical records, and administrative and Medicaid claims databases. One site contacted case-patients directly if a vaccination history was not found through an existing data source; self-reported vaccination histories were verified by contacting the vaccine provider, when possible.

Site-appropriate methods for obtaining screening rates in the catchment areas’ populations were investigated. Self-reported cervical cancer screening is subject to misclassification (especially overestimation); thus, novel methods to determine screening rates were explored at each EIP site, and methods using existing resources were developed to obtain screening estimates in 3 sites: California, New York, and Oregon. In California and Oregon, weighted estimates of screening were calculated by using available data from national and state-based surveys and administrative databases. National survey data indicated differences in screening rates between insured and uninsured women, so women in the catchment areas were divided into 2 groups on the basis of insurance status (insured or uninsured), and annual screening rates were obtained for each group by using data from the American Community Survey (http://www.census.gov/acs/www/) and the Behavioral Risk Factor Surveillance Survey (http://www.cdc.gov/brfss/data_documentation/index.htm) to estimate the relative proportion and the difference in screening rates between the groups. The insured and uninsured groups were then combined to estimate overall annual screening rates by age group (18–20, 21–29, and 30–39 years of age) adjusted for insurance status. New York obtained deidentified cervical cancer screening reports from cytopathology laboratories serving the catchment area. The reports, which were deduplicated within the laboratory, included patient age and results of the first screening in a given calendar year and combined and categorized into specified age groups. Screening rates were estimated by using data from the 2010 US census.

During 2008–2012, a total of 13,089 CIN2+ cases were reported among women >18 years of age. Almost half of reported cases (48.1%) were in women 21–29 years of age. Women in the youngest (18–20 years of age) and oldest (>50 years of age) age groups represented only 3.9% and 7.2%, respectively, of all cases (Table). HPV vaccination status was investigated for all women 18–39 years of age with CIN2+. Among those 18–30 years of age at the time of diagnosis who were eligible for vaccination before or during 2008–2012 (n = 7,344), a total of 3,621 (49.3%) had documented vaccination status in the medical chart or by self-report, and 894 (24.7%) of those women had initiated the vaccination series.

**Table:** Selected characteristics among women with a diagnosis of CIN2+, Emerging Infections Program HPV-IMPACT project, United States, 2008–2012*

Characteristic	No. (%)
Diagnosis age, y, N = 13,089	
18–20	507 (4)
21–29	6,294 (48)
30–39	3,774 (29)
40–49	1,575 (12)
>50	939 (7)
Race/ethnicity, N = 10,932	
White, non-Hispanic	6,629 (61)
Black, non-Hispanic	1,857 (17)
Hispanic	1,540 (14)
Asian	551 (5)
Other	355 (3)
Missing	2,157
Vaccination status, N = 7,344	
Vaccinated	1,811 (25)
Not vaccinated	1,812 (25)
Unknown	3,721 (51)
Not age-eligible	5,745
Diagnosis, N = 13,089	
CIN2	6,275 (48)
CIN2/3	2,149 (16)
CIN3/AIS	4,665 (36)
Site location, N = 13,089	
California	2,286 (17)
Connecticut	3,729 (28)
New York	2,813 (21)
Oregon	2,557 (20)
Tennessee	1,704 (13)

*AIS, adenocarcinoma in situ; CIN, cervical intraepithelial neoplasia; CIN+, CIN grade 2 or 3 or adenocarcinoma in situ; HPV, human papillomavirus.

Archived specimens were retrieved for 7,693 (72.2%) of 18- to 39-year-old women with a diagnosis of CIN2+ during 2008–2012. Of these specimens, 6,745 (87.7%) were histologically adequate and underwent DNA testing; HPV DNA was detected in 6,721 (99.6%) of the specimens. The prevalence of HPV types in CIN2+ lesions is shown in the Figure. Our findings confirmed that HPV-16 and -18 (i.e., types targeted by current vaccines) accounted for over half of all lesions in this population-based sample of women with CIN2+. DNA typing data from the project have also been helpful in predicting the effect of the new 9-valent HPV vaccine in the United States.

**Figure:**
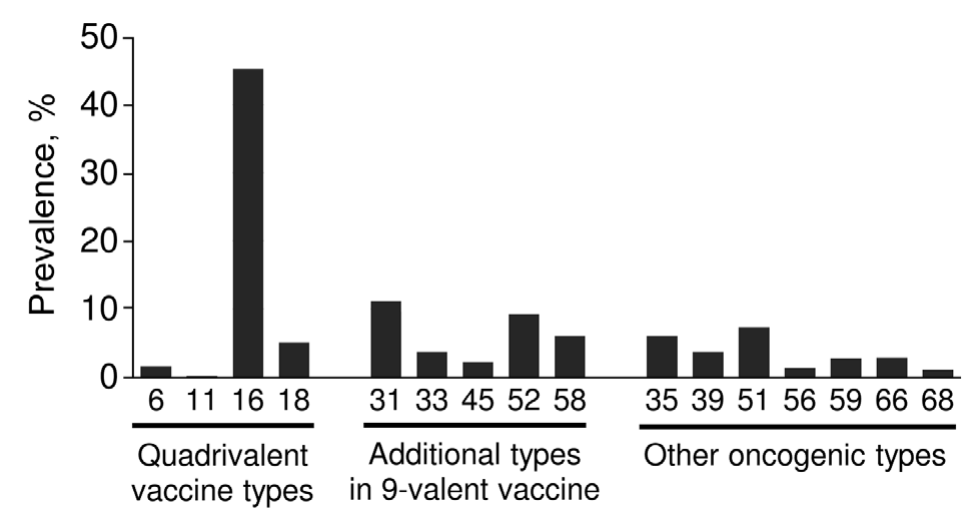
Prevalence of human papillomavirus (HPV) types among women with a diagnosis of cervical intraepithelial neoplasia grade 2 or 3 or adenocarcinoma in situ, Emerging Infections Program HPV-IMPACT project, 2008–2012. HPV-16 and -18 are the most common oncogenic HPV types; HPV-6 and -11 are nononcogenic HPV types that cause genital warts and respiratory papillomatosis.

Vaccination and screening history data were used to demonstrate that proportions of HPV-16– and HPV-18–attributable CIN2+ in women who initiated vaccination at least 24 months before receiving an abnormal screening test result were statistically significantly lower than proportions in unvaccinated women (*18*). Recent data indicate substantial declines in CIN2+ among women <25 years of age since HPV vaccine was introduced; not all of these declines can be explained by concurrent decreases in the use of screening (*19*). Investigation is ongoing to determine the extent to which the observed decreases may be attributable to vaccine effect and to further quantify vaccine effectiveness on type-specific lesions.

The HPV-IMPACT project has provided valuable information for monitoring the effect of HPV vaccine among the US population, including data on the type distribution of HPV before widespread introduction of the vaccine. A variety of challenges to developing a sustainable population-based system for monitoring CIN2+ and associated HPV types were identified and addressed during the project’s pilot phase. Since 2011, active surveillance has been ongoing at all 5 EIP sites, and the systems have been periodically evaluated. Laboratories serving the catchment areas are monitored through routine contact with health care providers, regional and local surveys, and other means to ensure completeness of reporting is maintained over time for all women in the catchment areas. Case ascertainment methods are continually updated and refined as the recommended diagnostic terminology evolves. New mechanisms for obtaining complete vaccination histories, such as interviewing case-patients and contacting vaccine providers to verify self-reported vaccination, are being explored. Efforts are ongoing to improve methods for measuring cervical cancer screening rates.

The success of the HPV-IMPACT project exemplifies the flexibility of the EIP network to expand core activities to include emerging surveillance needs beyond acute infectious diseases. Results from this project contribute key information on the effect of HPV vaccination among the US population.
